# Changes in oral pathology teaching methods during the COVID-19 pandemic and the impact on student performance (oral pathology teaching changes during the COVID-19 pandemic)

**DOI:** 10.1016/j.jds.2023.05.035

**Published:** 2023-06-14

**Authors:** Jin-Hao Cui, Chihoko Ikeda, Katsuhiro Suzuki, Harumi Isono, Yukino Hisano, Yuma Yamamoto, Takehiro Yoshikane, Tomoharu Okamura, Kazuya Tominaga

**Affiliations:** a Graduate School of Dentistry (Pathology), Osaka Dental University, Osaka, Japan; b Department of Oral Pathology, Osaka Dental University, Osaka, Japan

**Keywords:** Oral pathology, COVID-19, Teaching methods, Light microscopy, Virtual microscopy, Online learning

## Abstract

**Background/purpose:**

Coronavirus disease 2019 (COVID-19) has influenced the dental education in Osaka Dental University. The purpose of this study was to summarize the impact of COVID-19 on student performance and the current more appropriate teaching methods by comparing the changes in various oral pathology exam results before and after COVID-19.

**Materials and methods:**

The experimental and control groups consisted of second year students in the department of dentistry at our university for the years 2019 (136 people) and 2020 (125 people). The impact of different teaching methods on student performance was compared by calculating the mean scores and percentage of failures on various exams and whether or not class credits were earned between the two years. A *t* test was used to determine statistical significance.

**Results:**

The mean scores on the mini-tests were lower in 2020 than in 2019, while the average score of the intermediate exam and the number of students receiving class credits were higher. The mean scores on the practical and unit exams were not statistically significant between the years, but the failure rate on both exams was higher in 2019 than in 2020.

**Conclusion:**

COVID-19 had impacts on student performance. A comparison of the mean scores on the exams revealed that the use of microscopy, oral questions, and online animations contributed to improved performance on different exams. Therefore, to promote students' understanding and retention of memorized knowledge of oral pathology, the use of microscopes will be resumed whenever possible, as well as continuation with oral questions and online animations.

## Introduction

At the end of 2019, a novel coronavirus that causes severe acute respiratory syndrome coronavirus-2-related disease, called coronavirus disease-2019 (COVID-19), was reported in Wuhan City, Hubei Province, China.^1^, ^2^, ^3^, ^4^ According to the World Health Organization (WHO), the mortality rate from COVID-19 was 4.3% early in the pandemic.^5^ Since human-to-human transmission of the virus (including direct contact and air droplets) has been confirmed, in order to prevent the spread of COVID-19, the WHO recommends that countries take certain isolation measures to limit contact between infected and healthy individuals to control the global spread of the pandemic.^6^, ^7^, ^8^, ^9^ Since the first case in Japan in January 2020, the country has had a total of more than 32 million infected persons by March 2023.^10^ About one in four people have suffered from the disease. In response to the sudden outbreak, the Japanese government issued an emergency declaration on April 7, 2020, which included wearing masks, closing schools, requesting that the number of people dining or drinking together as a group to be limited to four per table and so on.^11^ Relevant defensive measures have also been developed. Basic infection control measures include avoidance of the “Three Cs”, 1) Closed spaces (with poor ventilation), 2) Crowded places (with many people), and 3) Close-contact settings (with conversation or speech at arm's reach), maintaining distance from others, wearing masks, hand washing and other hand hygiene, and ventilation.^12^ All students at Osaka Dental University were restricted from going to school for 2 months. All club activities were also stopped.

**Figure 1:**
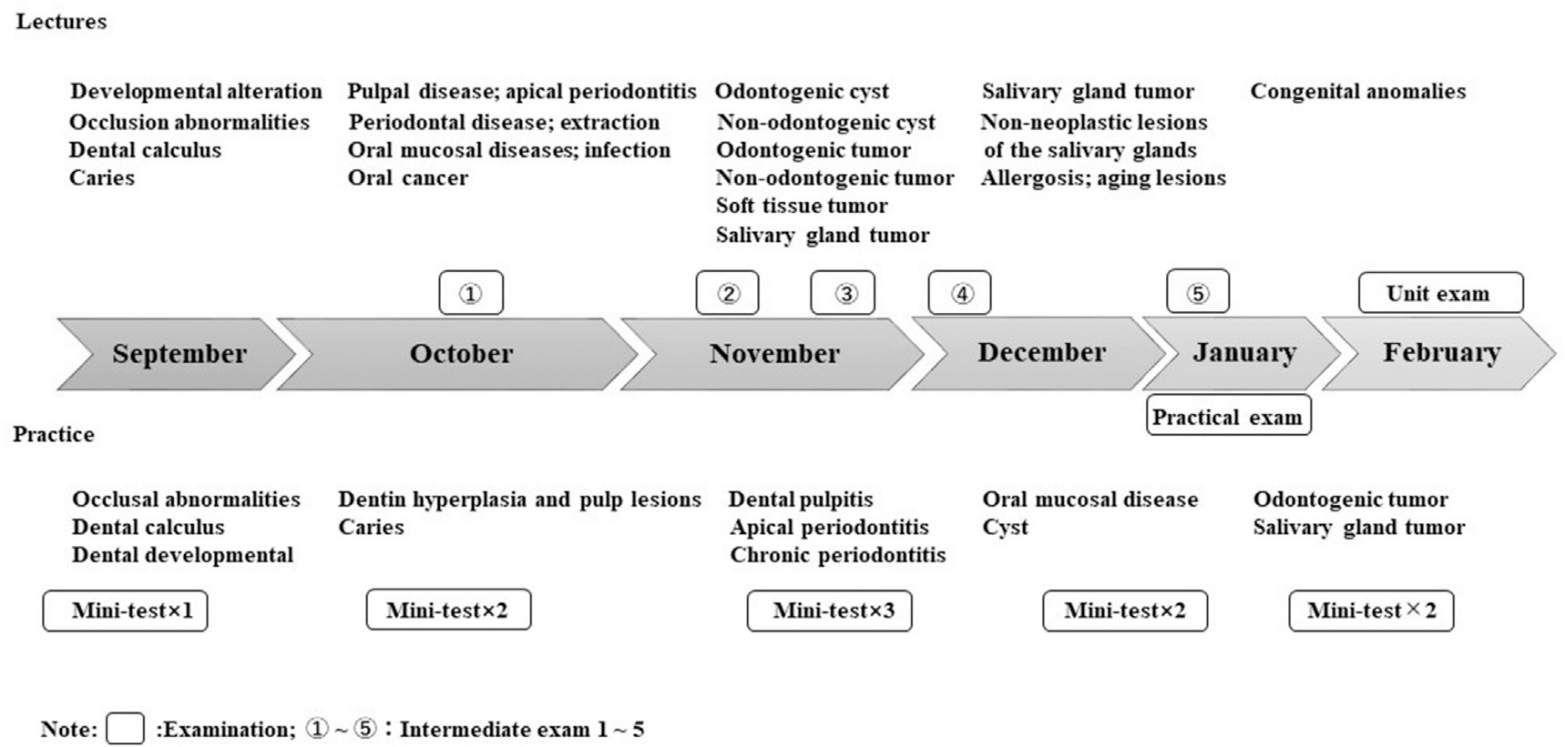
Schedule of lectures, practice, and various exams for the year 2020.

Dental education differs from other disciplines in that, in addition to basic knowledge, much emphasis is placed on clinical skills training.^9^ At our university, lectures and practice in oral pathology are given from September to February of the following year of the second academic year. Topics include dental developmental abnormalities, caries, pulpal periapical disease, periodontitis, precancerous lesions, cysts and tumors (Fig. 1). In the lectures and practice, students study the gross and microscopic findings of various diseases in the oral area, and in practical training, they use light microscopy to understand the characteristics of various diseases and acquire knowledge that leads to histopathological diagnosis (Fig. 1). After COVID-19, the oral pathology practice class for second year students was changed from one microscope-practice room to two large lecture rooms according to student ID number. The lectures and the explanation of all practical contents were given by distributing pre-learning video animations. In our university, there are two main forms of examinations for oral pathology practices, the mini-test and the practical exam, both of which are based on the identification of histopathological pictures. Knowledge from oral pathology lectures is tested mainly in the intermediate and unit exams. Questions in text form are the main focus.

This study aimed to compare the changes in teaching methods before and after COVID-19 and whether they affected students' performance on various tests, and finally to explore teaching methods that are appropriate for the current situation.

## Materials and methods

### Participants

The participants of this study were all second year students of the department of dentistry in the 2019 (136 people) and 2020 (125 people) academic years at Osaka Dental University. In the two different academic years in this report, there were no changes in the instructors for the oral pathology course. There were no changes in the course content or objectives for either course. The content of the practical training and the 10 single-choice mini-tests following each practice was identical for both academic years, as was the format and scope of the questions asked on the intermediate exam, practical exam and unit exam.

### Types of exams

Each mini-test has 10 single-choice questions, each with a 40-s response time. The answers and explanations are given after the mark sheets have been turned in. The practical exam evaluates the level of proficiency gained in practical training by having the examinee answer a question-and-answer passage on the histological diagnosis, findings or histopathological image based on images of the specimens studied in the practical training. It takes about 90–120 min depending on the situation. Intermediate exams are administered five times during the oral pathology lectures. Each exam includes two 10-min tests and 25-min of self-study between the two tests. Since 2019, the exam has been a written exam. Unit exams are comprehensive exams at the end of all courses, consisting of multiple-choice, fill-in-the-blank, and expository questions. They take about 70 min. Class credits are given based on a combined evaluation that calculates the percentages for the submission of class notebooks and each type of exam (excluding mini-test).

### Calculation of grades

**Figure 2:**
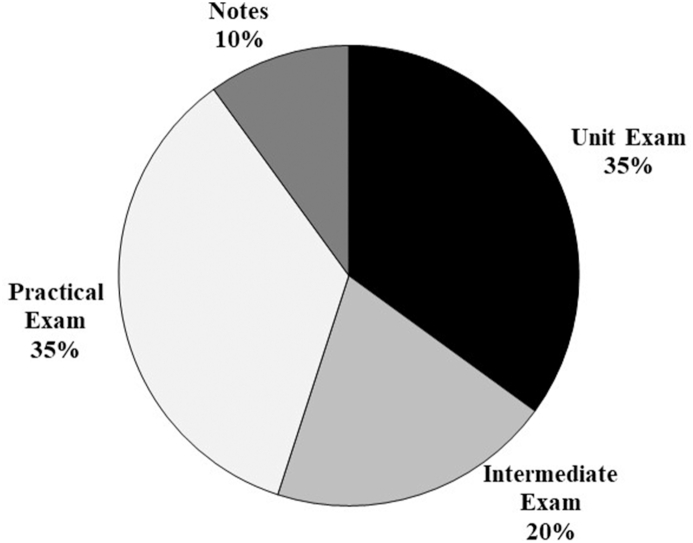
Evaluation methods and items.

The calculation of the average scores on the various examinations, class credits given, and the criteria for determining failure are as follows: (1) mini-test: the average of 10-time average points (the average points for each time: the sum of the scored points divided by the total number of people). (2) Intermediate exam: average points as a percentage of the full points. The number of full points was 50 in 2019 and 40 in 2020. (3) Practical exam and unit exam: the sum of the scored points divided by the total number of students.65 points and above is a passing grade. (4) The method of conferring class credits and calculating the percentage of each item is shown (Fig. 2). Round up to the nearest whole number if greater than 64 points. Exactly the same for both years.

### Process and methods of the oral pathology practice

**Figure 3:**
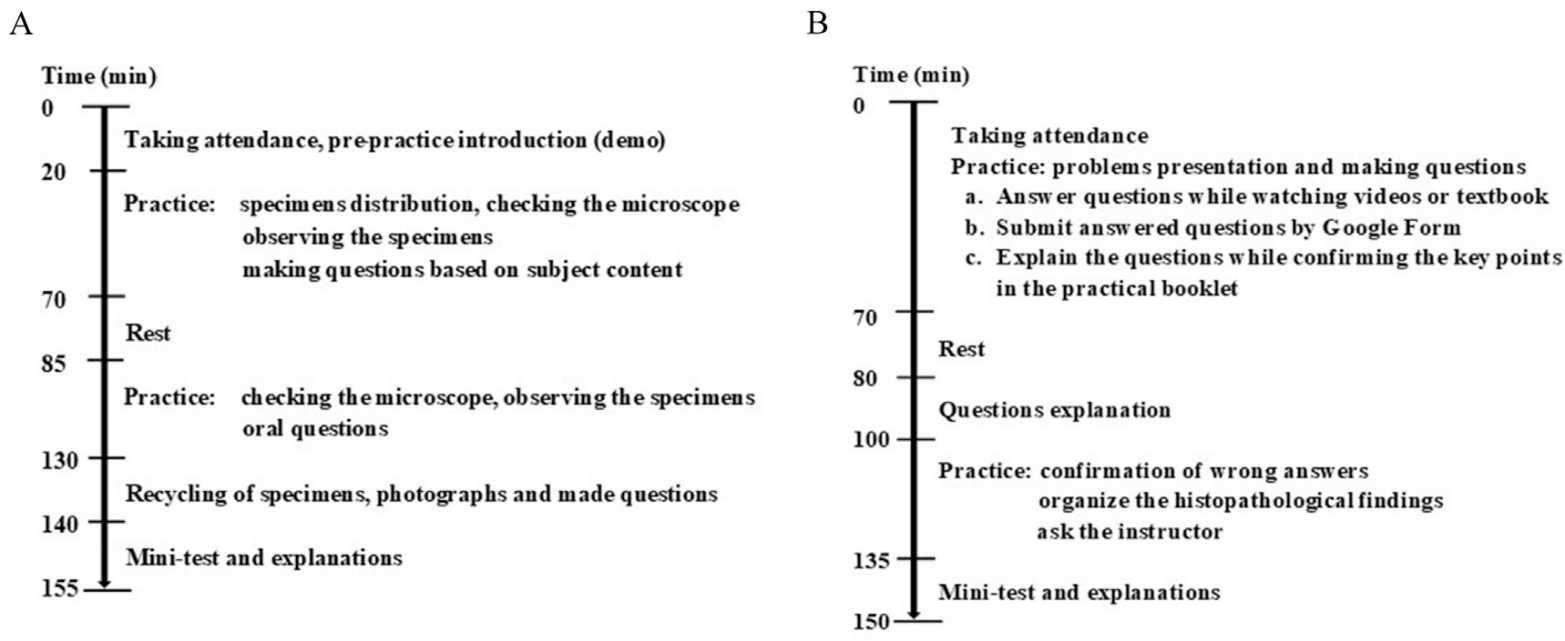
Two years of oral pathology practice process and teaching methods. (A) 2019, and (B) 2020.

In 2019, after confirming the list of attendees, specimens were distributed and the findings of the tissues were drawn in the lab book while the specimens were observed under microscope. After the break, the instructor asked oral questions in order, and finally, there was a mini-test and explanations (Fig. 3A). In 2020, after confirming the attendance list, each student watched a narrated video uploaded to YouTube on the content of the practice, answered questions while reviewing the textbook and their own notes, and submitted answers by Google Forms. The findings of the tissues were drawn in the lab book. After a break, the instructor answered questions. Finally, there was a mini-test and explanations (Fig. 3B).

### Statistical analysis

All data collected were stored in Excel (ver.16.32) and used for statistical analysis. The differences in the mean scores for various investigated items were compared between 2019 and 2020 by Student's *t* test. The result was significant if the *P-value* was less than 0.05.

## Results

**Figure 4:**
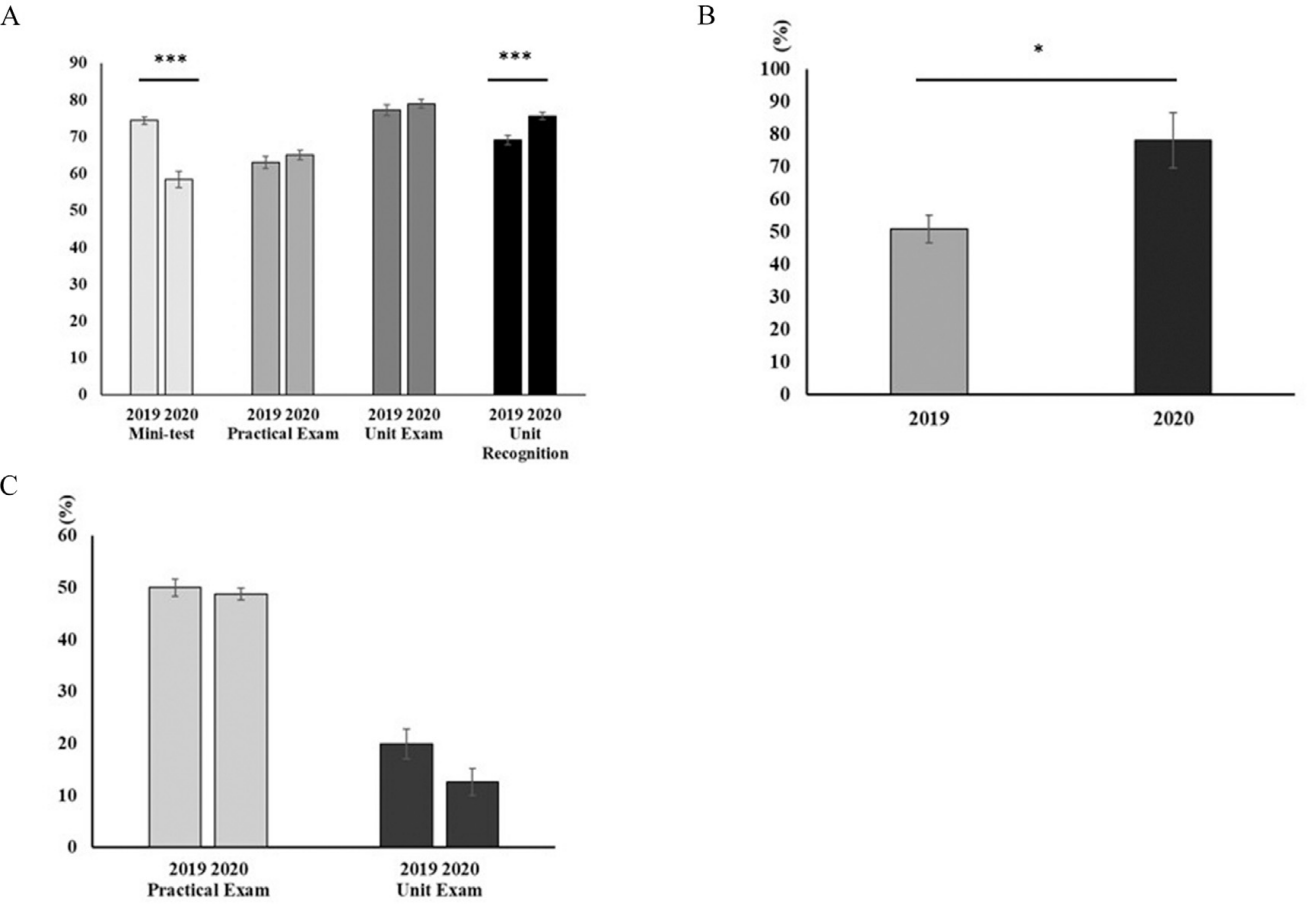
Results of various exams and class credits for the 2 years. (A) Average scores and class credits. (B) Average score on intermediate exam. (C) Failure rate on practical and unit exams. ∗: *P* < 0.05; ∗∗∗: *P* < 0.001.

**Figure 5:**
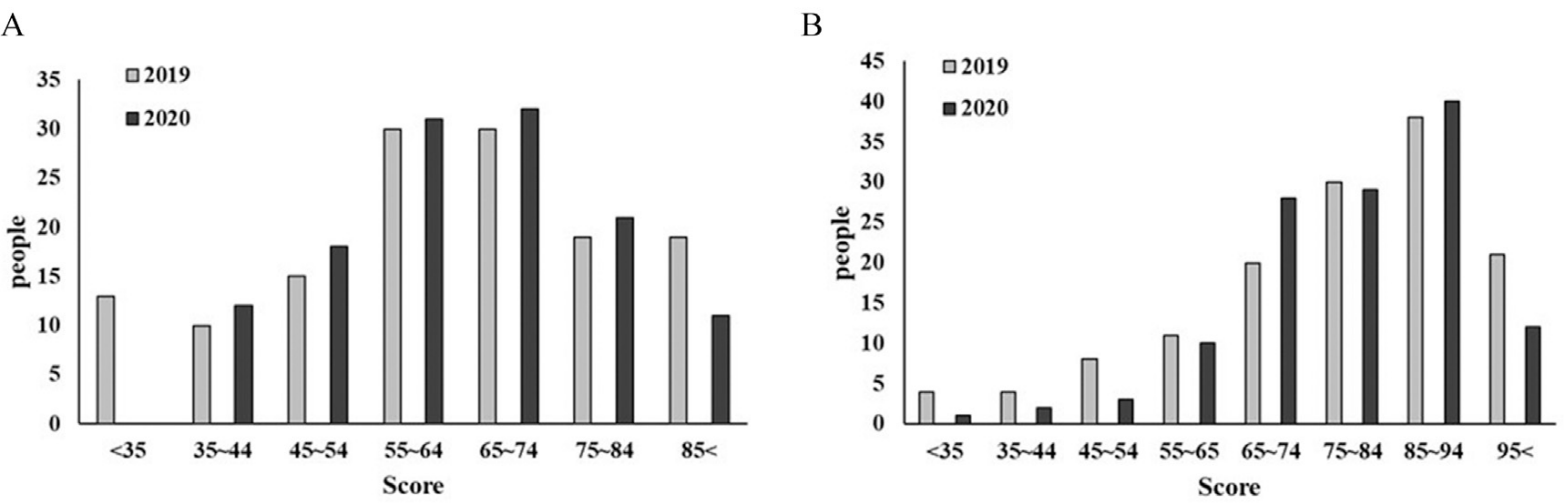
The distribution of scores for the two years. (A) Practical exam (B) unit exam.

The average score for the microscopy and oral questions on the mini-test in 2019, prior to COVID-19, was significantly higher, at 74.4, than in 2020, at 58.4 (Fig. 4A). On the intermediate exams, the average score was significantly lower in 2019 than in 2020 (Fig. 4B). Class credits were earned by 69.2 in 2019, lower than 75.7 in 2020, which was a statistically significant difference. The average scores on the practical and unit exams for the two years was not very different, but slightly higher in 2020 than in 2019 (Fig. 4A), as was the failure rate. Neither difference was statistically significant (Fig. 4C). However, in terms of the distribution of points scored, the lowest score on the practical exam was higher in 2020 than in 2019 (Fig. 5A). Scores of 65–95 points on the unit exam were also higher in 2020 (Fig. 5B).

## Discussion

The average scores on the mini-tests in 2020 were lower than in 2019, when there were the photos of tissue sections, microscopy practice and oral questions. The oral questions asked the diagnostic names of various oral diseases and their characteristic cells, reactions, and other information using histopathological sections. The photos were pictures of more typical images of each oral disease with a description of the characteristic cells and manifestations of that disease. For example, the photo of dentinal caries indicated the characteristics of the layers of caries, carious cone and caries crack. The mini-tests after the practice were mostly based on pathological tissue sections, and a greater visual understanding of the morphology and characteristics of cells and tissues could be obtained with observation under the microscope. The paper photos further reinforced students' memory, so they could answer more questions correctly on the mini-test immediately after the practice class. Conversely, the practical exams, which are also based on the correct identification of histopathological pictures of oral diseases, had lower average scores and higher failure rates in 2019 than in 2020. Perhaps the prolonged use of microscopy caused visual fatigue. According to one study, the most common occupational concerns of microscope users were musculoskeletal problems of the neck and back regions, eye fatigue, stress due to long hours of use, and anxiety during or after microscopy use.^13^ At the same time, glass specimens were available for viewing in the oral pathology research laboratory, but almost no students came. On the unit exam, although the average scores for the two years were relatively close, the high failure rate in 2019 and the results of the intermediate exam showed that the advantages of the microscopy were even less obvious in exams that were mainly in the form of text-based questions. The intermediate exam in 2018 consisted of 50 multiple choice questions with an exam time of 50 min. In 2019, the exam type was changed to a written exam. It consisted of a 10-min exam, followed by 25 min of self-study, and then another 10-min exam with different questions in the same range.^14^ Student average scores on the intermediate exam also improved compared to 2018.

In 2019, when there was no COVID-19, students in the physical class could review textbooks and notes. They could ask questions in class or come to the oral pathology research lab after class. In 2020, due to COVID-19, we had to change to online lectures and animations which students could watch at home repeatedly to enhance their memory. This is perhaps one of the objective reasons for the differences seen. According to a study conducted by the Japanese Ministry of Education, Culture, Sports, Science and Technology, students' (national and public universities, private universities and colleges of technology in Japan) satisfaction with online learning was high for 56.9% of those surveyed, with response of the advantage of a freer place to study accounting for 66.1%.^15^ In a study on anatomical teaching methods, Nedžad Hadžiomerović et al. also showed that online teaching, such as video lectures, can improve students' performance and contribute to long-term memory of knowledge.^16^ The study attitude of each class, which is a unitive factor, also affected the final result of the exam. Another thing that must be mentioned is that most students in Japan mainly commute by train or subway. According to a survey conducted by the Japan Student Services Organization, more than 80% of students (undergraduate, junior college, and graduate students, but excluding correspondence courses, students on leave of absence, and international students) who live at home have a one-way commute of 30 min or more to school.^17^ This means that second-year students in 2019 before Covid-19 spent more time than the 2020 students in commuting to and from school, and the exhausting commute may have affected concentration in class. Some studies have shown that many working adults and students in Japan who commute to work and school on the same routes every day are continuously and intermittently exposed to similar stressor stimuli every day, and continued exposure to such stimuli can lead to the onset of stress reactions, resulting in effects such as decreased performance in work and learning.^18^ In addition, in a study of total health conditions and commute time in university and junior college students, it was shown that long commutes have a negative impact on mental and physical health.^19^ Students in the year 2020, on the other hand, were able to complete their courses other than their practice classes at home with relative ease and freedom of time, and may have been more efficient in their studies. The results for class credits also support this inference.

Although online learning has considerable advantages, the above survey showed that 44.7% of students (national and public universities, private universities and colleges of technology in Japan) still find it more difficult to understand the course content in online courses than in in-person, and 53% of them feel lonely not being able to study with their classmates.^15^ The current technology is fully capable of handling large-scale online teaching, but it also places higher demands on teachers, including the use of various software and online communication with students.^20^

Our university also still uses traditional light microscopy. This has some shortcomings, such as the inconvenience of handling equipment and materials, limited field of view, and storage and preparation of glass specimens, among others. Some studies have shown that the digital virtual microscope, which has emerged in recent years, is more popular with the majority of students. This technology uses a computer to analyze glass slide specimens. After traditional glass slides are prepared, they are scanned and digitized in high resolution so that they can be interpreted and analyzed with specific software.^21^ These systems are easy to operate, images are easy to store, and they are in line with the current climate of advances in computer technology. Nevertheless, light microscopy is also used in basic research and in most hospital pathology diagnostics. Storing and uploading virtual slides can also take some time.^22^^,^^23^ Therefore, the light microscope cannot be completely eliminated from dental education. Maybe in the future the two kinds of microscopy can be used in combination for teaching.

In addition, a limitation of this study is that there was no systematic implementation of questionnaires for students in either academic year, so we are unable to provide better evidence for the association between general studies and grades. That is our next research plan.

In conclusion, the use of online animations does seem to help students improve their oral pathology exam scores, while the learning of optical microscopy techniques as the most basic tool for pathology course learning and clinical diagnosis cannot be neglected. Oral questions in the practical class are one opportunity for one-on-one student-teacher interaction. The use of histology pamphlets helps to consolidate the student's knowledge. Students can also ask the instructor immediately if they have any questions, like pathologic features of a disease or differential diagnosis. From the period of severe restrictions in April 2022, such as no club activities, no gathering for meals and so on, to the gradual decrease in the number of infections into 2023, teachers have been required to adjust their teaching methods according to the situation at the time.

## Declaration of competing interest

The authors have no conflicts of interest relevant to this article.
